# A Novel Molten Salt Mediated Synthesis of Mesoporous Metal Oxides with High Crystallization

**DOI:** 10.1021/acscentsci.3c01462

**Published:** 2024-02-26

**Authors:** Dongsheng Ma, Hanpeng Lu, Yu Zhou, Shuaihu Jiang, Duan Wang, Qin Yue

**Affiliations:** † Institute of Fundamental and Frontier Sciences, University of Electronic Science and Technology of China, Chengdu 610054, China; ‡ Orthopedic Research Institution, Department of Orthopedics, West China Hospital, Sichuan University, Chengdu 610041, China

## Abstract

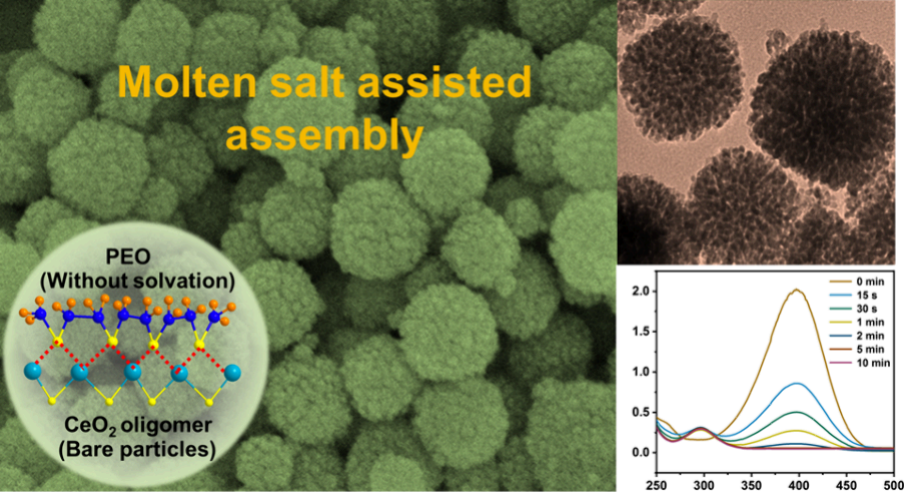

The controlled synthesis of mesoporous metal oxides remains a great challenge because the uncontrolled assembly process and high-temperature crystallization can easily destroy the mesostructure. Herein, we develop a facile, versatile, low-cost, and controllable molten salt assisted assembly strategy to synthesize mesoporous metal oxides (e.g., CeO_2_, ZrO_2_, SnO_2_, Li_2_TiO_3_) with high surface area (115–155 m^2^/g) and uniform mesopore size (3.0 nm). We find this molten salt mediated assembly enables the desolvation of the precursors and forms bare metal ions, enhances their coordination interaction with the surfactant, and promotes their assembly into a mesostructure. Furthermore, the molten salt assisted crystallization process can lower the collision probability of the target metal atom, inhibit its further growth into large crystals, and achieve a well-maintained mesostructure with high crystallization. Furthermore, this method can be expanded to synthesize various structured mesoporous metal oxides, including hollow spheres, nanotubes, and nanosheets by introducing the carbon template. The obtained mesoporous CeO_2_ microspheres loaded with Cu species exhibit excellent antibacterial performance and superior catalytic activity for the hydrogenation of nitrophenol with high conversion and cycling stability.

## Abstract 2

A facile and versatile molten salt-assisted assembly strategy toward highly crystallized mesoporous metal oxide for efficient antibacterial and hydrogenation catalysis is described.

## Introduction

Mesoporous material, a type of porous material with a pore size between 2 and 50 nm, possesses a high surface area, uniform pore size, large pore volume, various mesostructures, and tunable composites.^1−6^ As a result, mesoporous materials exhibit wide applications in biomedicine, environmental remediation, catalysis, sensors, and energy storage and conversion.^7−9^ A majority of mesoporous materials are silica-based or polymer derived carbon-based composites. Metal oxides possess the properties of a semiconductor with a particular band gap and high crystallinity, and are potentially applied in photocatalysis, gas sensing, photothermal therapy, and catalyst carriers. Mesoporous metal oxides with high porosity and large exposure of surface unsaturated metal atoms can definitely enhance their properties and thus improve their performance.^10−15^ However, the design synthesis of mesoporous metal oxides with high crystallization is a great challenge because the high-temperature crystallization usually inevitably induces mesostructure collapse.

The typical synthesis of mesoporous metal oxides usually includes two main steps: the formation of mesostructure using a metal precursor and template (hard-template or soft-template) and the subsequent high-temperature crystallization as well as template removal.^16−20^ The hard-template method, similar to nanocasting, starts with infiltrating precursors into ordered mesoporous carbon or mesoporous silica (MCM-41, KIT-6, SBA-15) and then etching off the template to obtain the inverse mesostructure.^21^ Gu et al.^22^ synthesized mesoporous ZrO_2_ as a hard template, in which the surface-modified functional groups help to enhance the interaction between the template and the precursor. Unfortunately, the hard template method usually requires complicated procedures with the inevitable waste of nonrecyclable templates, and the difficulty in the adjustment of mesopores and morphology, and is unable to be used in large-scale synthesis. In contrast, the soft template strategy, primarily evaporation-induced self-assembly (EISA), achieved through the evaporation of the precursor/surfactant/ethanol and formation of lyotropic liquid crystalline (LLC) driven by the coordination interaction between the surfactant and metal cation, is a flexible synthesis technique to obtain ordered mesostructures.^23−25^ However, the mesoporous framework usually undergoes collapse due to atom rearrangement in the subsequent high-temperature crystallization process, producing nonporous metal oxide with large crystal particles. To address the problem, Feng et al.^26^ proposed a carbon residues supporting strategy by adopting an amphiphilic block copolymer with sp^2^ hybridized benzene ring-involved segments (e.g., PEO-*b*-PS), which possesses high thermal stability as the soft template to help stabilize the mesostructure. Liu et al.^27^ developed the synthesis of two-dimensional (2D) single-layer ordered mesoporous oxides (TiO_2_, CeO_2_, Al_2_O_3_, ZrO_2_, etc.) by using solvent volatilization to induce domain-limited coassembly of the block copolymer with the precursor on the surface of NaCl crystals. Despite the progress in the synthesis of mesoporous metal oxide, the existing method has difficulties in controlling the assembly process due to the humidity sensitivity and rapid hydrolysis rate of metal precursors,^28^ achieving highly crystallized mesoporous metal oxides and large-scale synthesis. Molten salts are soluble, stable, and recoverable high-temperature solvents that not only accelerate the reaction process, but also facilitate the desolvation of precursors to form bare ions.^29^ Wang et. al.^30^ developed a molten salt supersolubilizing method to prepare Co_2_Mo_3_O_8_ nanoplates for Li–S batteries. Dag et al.^31,32^ spin-coated a hydrated metal salt/LiNO_3_/surfactant/ethanol mixture to form a lyotropic liquid crystalline (LLC) film and transformed it into mesoporous metal titanates by a calcination step. Hence, exploiting a facile, low-cost, and general strategy that enables the synthesis of homogeneous mesoporous metal oxides with high crystallization is significant and urgently required.

Herein, we have developed a facile and general molten salt assisted assembly method to prepare mesoporous metal oxides through combining a solvent-free self-assembly and crystallization in one step. The molten salts are a meltable, stable, and recoverable medium at their eutectic point. Different from an organic solvent-involved strategy, the molten salts method exhibits advantages and properties as follows: (1) the molten salts can facilitate the desolvation of precursors to form bare metal ions, which are directly coordinated to the block copolymer micelles, thus greatly enhancing the interaction between the metal ions and the ether bonds of the PEO blocks and boosting the assembly of mesostructures; (2) the molten salt-assisted crystallization process provides a liquid-heating microenvironment with much interference ions, where the collision probability of the target metal atom is much lower than the traditional solid powder calcination. It efficiently inhibits the further growth of large crystals and maintains the mesostructures during the high-temperature crystallization process; (3) the facile synthesis allows the assembly, crystallization, and removal of surfactant in one step, not only avoiding the utilization and waste of organic solvents but also largely simplifying the separation and purification process, lowering the cost, and promoting large-scale synthesis. As a result, mesoporous CeO_2_, ZrO_2_, SnO_2_, and Li_2_TiO_3_ were successfully synthesized using this method. Furthermore, this strategy can be extended to synthesize hollow structured mesoporous metal oxides through introducing a carbon template with various morphologies (e.g., microspheres, nanofibers, and nanosheets). Notably, mesoporous CeO_2_ with hollow microspheres, hollow nanotubes, and nanosheets were fabricated. Due to the dispersed active sites and large surface area, the mesoporous CeO_2_ microspheres loaded with Cu species (Cu-mCeO_2_) show a strong antibacterial ability toward *Escherichia coli* and *Staphylococcus aureus*. Furthermore, Cu-mCeO_2_ exhibits superior catalytic performance toward the hydrogenation of nitrophenol with high efficiency and stability. This work brings new insights into the design synthesis of mesoporous metal oxides with high surface area and high crystallization with wide and potential applications.

## Results and Discussion

**Figure 1:**
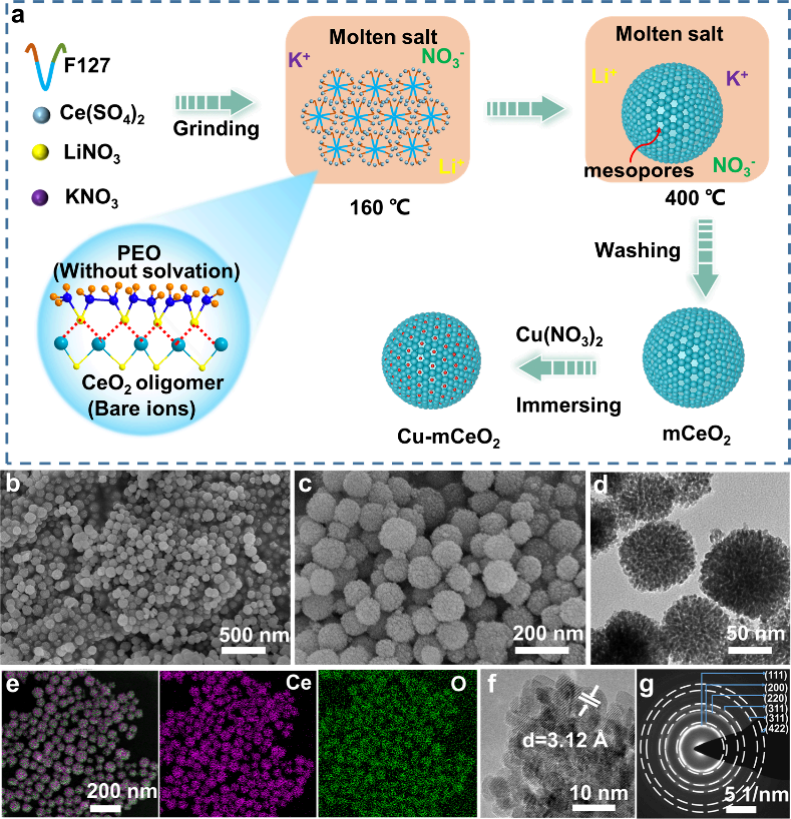
(a) Scheme of the synthesis procedures for mCeO_2_ microspheres by a molten salt-assisted self-assembly strategy; (b, c) SEM images of mCeO_2_ with different magnifications; (d) TEM, (e) EDS-mapping, (f) HRTEM, and (g) SAED pattern images of mCeO_2_.

Figure 1a illustrates the synthesis process of mCeO_2_. The Ce(SO_4_)_2_, block copolymer F127, and nitrate are ground to homogeneously mix and then heated to 160 °C in air. Due to its low eutectic temperature (123 °C) and excellent chemical stability (Figure S1), the mixed nitrate (LiNO_3_-KNO_3_) gradually melts into a stable liquid that acts as a reaction medium. The surfactant F127 tends to aggregate into micelles in the hydrophilic molten salt and assemble with Ce^4+^ driven by the coordination interaction between Ce^4+^ and the ether oxygen derived from F127 micelles. Then, the mesostructured organic–inorganic hybrid complexes are formed in the molten salt assisted assembly process. As the temperature is further increased to 400 °C, CeO_2_ begins to crystallize in the molten salt, and the surfactant F127 gradually decomposes, generating the mesoporous structure. It not only serves as the reaction medium to induce the assembly process, but it also provides a high-temperature ion liquid environment for the crystallization process (liquid phase crystallization process). As known, the traditional crystallization through directly heating solid powder usually leads to the rapid migration and rearrangement of the metal atom, and the collapse of the mesostructure. Different from that, the liquid phase crystallization process in molten salt inhibist the further growth into large CeO_2_ crystals due to the interference of other molten salt ions in the liquid. As a result, the mesoporous structure is retained as well as a high crystallization degree. Finally, the extra solidified salt is completely removed by washing with water, thus obtaining highly crystallized mesoporous CeO_2_.

**Figure 2:**
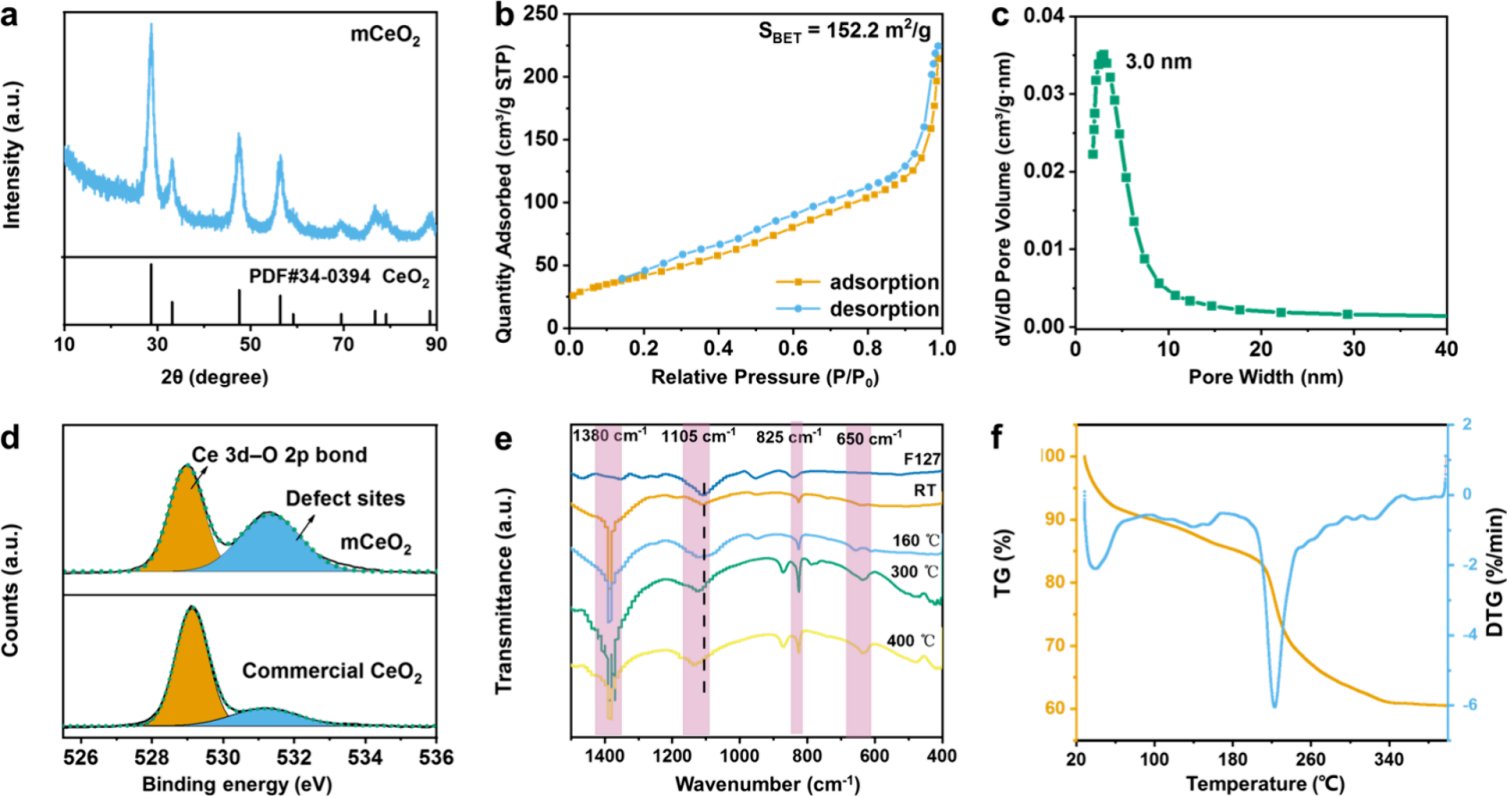
(a) The XRD pattern of mCeO_2_; (b) nitrogen adsorption and desorption isotherm and (c) pore size distribution of mCeO_2_; (d) O 1s XPS spectra of mCeO_2_ and commercial CeO_2_; (e) infrared spectra of F127 and CeO_2_ in molten salt at different temperatures; (f) thermogravimetric curve of mCeO_2_.

The scanning electron microscope (SEM) images (Figure 1b,c) of the obtained mCeO_2_ exhibit a uniform spherical morphology with a diameter of 120 nm, and the uniform mesopores over the microspheres are clearly observed from the transmission electron microscope (TEM) image (Figure 1d). The corresponding energy dispersive X-ray spectroscopy (EDS) elemental mapping (Figure 1e) confirms the uniform distribution of Ce and O over the whole microspheres, and the elemental ratio of O/Ce is about 2:1. X-ray diffraction (XRD) pattern (Figure 2a) matches well with the standard PDF#34-0349, indicating a pure cubic fluorite phase for mCeO_2_ microspheres. The electron diffraction and lattice stripe analysis of the selected area (Figure 1f,g) reflect a cubic structured CeO_2_ phase with a lattice spacing of 3.12 Å corresponding to the (111) crystal plane. N_2_ adsorption–desorption isotherm analysis was conducted to determine the surface area and pore parameters. The mCeO_2_ spheres (Figure 2b) show characteristic type-IV curves with H2-type hysteresis lines, indicating the presence of mesopores. The surface area was calculated, based on the Brunauer–Emmett–Teller (BET) method, to be 152.2 m^2^/g, which is 15 times that of commercial CeO_2_ (Figure S2). Due to the desolvation effect, the formed micelle in molten salt is definitely much smaller than that in aqueous/alcohol solution. The pore size distribution curve, derived from the desorption branch using the Barrett–Joyner–Halenda model, reveals that mCeO_2_ spheres (Figure 2c) possess a uniform pore diameter of 3.0 nm.

X-ray photoelectron spectroscopy (XPS) was utilized to characterize the chemical composition and valence states of mCeO_2_. Compared to the commercial CeO_2_, mCeO_2_ has a higher proportion of oxygen defects (Figure 2d) with the high concentration of Ce^3+^ (907 eV, 885 eV) in the Ce 3d spectra (Figure S3).^33^ The higher oxygen defect concentrations are also reflected in the Fourier Transform infrared spectroscopy (FTIR) and Raman spectra. In the Raman spectrum of CeO_2_, the oxygen vacancies on the surface can be compared by the intensity ratio of the D-band and F2g peaks (*I*_D_/*I*_F2g_), and one can see from Figure S4a that the concentration of oxygen vacancies in CeO_2_ is higher compared to that of commercial CeO_2_.^34^ Due to the higher concentration of oxygen vacancies, a higher concentration of water molecules is adsorbed on its surface, which leads to the appearance of obvious O–H vibrational peaks in the infrared spectra of mCeO_2_^35^ (Figure S4b). The oxygen vacancies mainly come from the insufficient oxidation from an oxygen-deficient environment in the molten salt. Moreover, according to the thermogravimetric curve (Figure S5), the trace residual carbon (∼4 wt %) derived from the block copolymer may account for the high oxygen defect concentration. The high surface area and defect concentration are favorable for effective loading of guests and further enhancing the applications in catalysis, etc.

To deeply understand the assembly mechanism in the molten salt assisted assembly process, the surfactant F127 was replaced by PVP and CTAC in the synthesis. As shown in Figure S6, nonuniform large CeO_2_ particles were formed without distinct mesopores. It is speculated F127 plays a key role in the morphology control and mesoporous structure formation in the molten salt assisted assembly process. F127, a nonionic surfactant, is a triblock copolymer consisting of PEO-PPO-PEO. In the normal evaporation induced self-assembly (EISA) process, the stronger solvation effect of organic solvents hinders the interaction between F127 and metal precursors, which is not favorable for their assembly and the further formation and retention of the mesostructures. In this work, both the precursor and the molten salt are hydrophilic, whereas PEO is the hydrophilic segment and PPO is the hydrophobic segment in F127. Hence, a micelle with PPO as the inner core and PEO as the shell form in the molten salt, and the Ce^4+^ will aggregate at the PEO shell through the coordination interaction between Ce^4+^ and ether oxygen atom from EO segment. Moreover, without a “solvent effect” in the molten salt, the PEO block is directly coordinated with the bare Ce ions by ether bonds, effectively boosting the assembly process.^29^ This can be confirmed by the FTIR spectra of the sample at different stages. As shown in Figure 2e, four obvious peaks are observed: C–O–C (1105 cm^–1^) from F127, a nitrate vibrational peak (1380 cm^–1^, 825 cm^–1^) from LiNO_3_ and KNO_3_, and a sulfate vibrational peak (650 cm^–1^) from Ce(SO_4_)_2_.^36−38^ It is observed the corresponding ether bond vibration peak (C–O–C) has a significant red shift, confirming that the bare Ce ions in the molten salt have a strong coordination with PEO. The formation process of mCeO_2_ is traced by TEM and XRD analysis during different reaction stages (Figures S7–S8). It is observed the crystallinity of mCeO_2_ was gradually enhanced with the stronger diffraction lines.

Noteworthy, this molten salt assisted assembly method has good universality for the synthesis of other mesoporous metal oxides. As Ce(SO_4_)_2_ was replaced by Zr(SO_4_)_2_, SnCl_2_, and Ti(SO_4_)_2_ while other synthesis parameters remained, mesoporous ZrO_2_, SnO_2_, and Li_2_TiO_3_ were generated, respectively. One can see that they all have uniform open mesoporous channels from the TEM images (Figure S9). The XRD patterns of mesoporous ZrO_2_, SnO_2_, and Li_2_TiO_3_ exhibit high crystallinity that corresponds to the standard PDF#49-1642, PDF#71-0652, and PDF#75-1602, respectively. The mesoporous ZrO_2_, SnO_2_, and Li_2_TiO_3_ have large surface areas of 77 m^2^/g, 83 m^2^/g, and 193 m^2^/g, and a homogeneous pore size of 3.5, 3.8, and 3.8 nm, respectively, according to nitrogen adsorption and desorption analysis (Figure S10). This suggests that the ether bond of F127 has a strong coordination ability for bare Zr, Sn, and Ti ions, which considerably contributes to the mesostructure assembly in the molten salt environment. More importantly, mesoporous high-entropy oxide (CeZrTiSnCoNi)O_*x*_ can be synthesized with this molten salt assisted assembly method. EDS mapping (Figure S11a) confirms the uniform distribution of Ce, Zr, Ti, Sn, Co, and Ni elements for the mesoporous (CeZrTiSnCoNi)O_*x*_. The mesoporous high-entropy oxide has a large surface area of 178 m^2^/g and a uniform mesopore size of 3.6 nm (Figure S11c,d). This method is not only facile and suitable for g-scale synthesis (Figure S12) but also is versatile for the fabrication of a wide range of mesoporous metal oxides to meet diverse applications.

**Figure 3:**
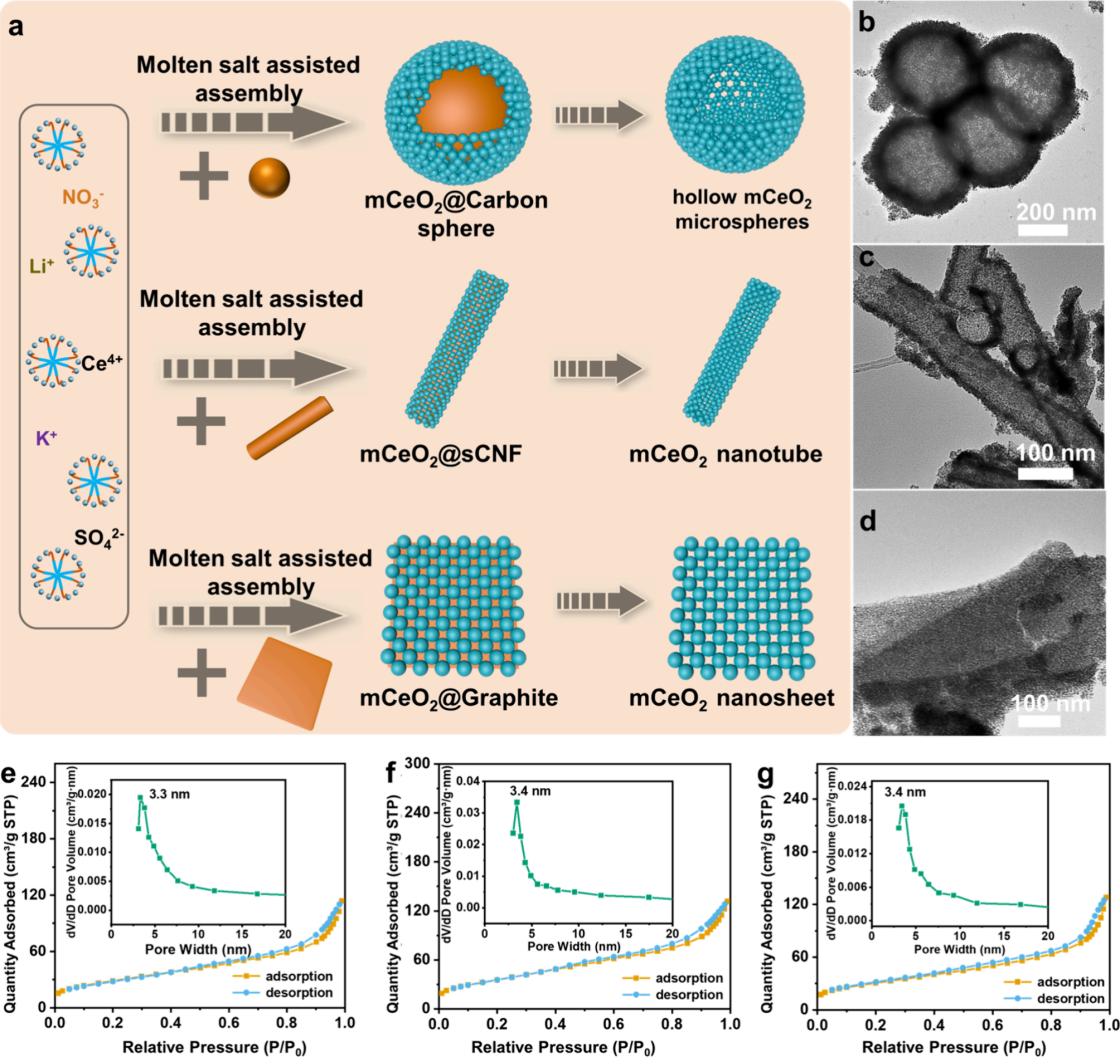
(a) Scheme for the synthesis of mCeO_2_ with various morphologies; TEM images, and nitrogen adsorption and desorption isotherm of (b, e) mCeO_2_ hollow microspheres, (c, f) mCeO_2_ nanotubes, and (d, g) mCeO_2_ nanosheets. The insets in (e−g) are the corresponding pore size distribution curves.

To further investigate the structure regulation for this molten salt assisted assembly method, carbon materials with various morphology including microspheres and nanofibers (Figure S13–S14, Figure 3a), that served as a hard template, were introduced into the reaction medium for the synthesis of mCeO_2_. At the first stage at 160 °C, the assembly of the F127 micelles/CeO_2_ hybrids happen at the surface of the carbon, and it forms a composite structure with carbon as the core and mCeO_2_ as the shell. With further high temperature treatment (400 °C), the carbon directly volatilized, generating mesoporous CeO_2_ hollow spheres (Figure 3b) and nanotubes (Figure 3c). Furthermore, two-dimensional mesoporous CeO_2_ nanosheets (Figure 3d) can be successfully obtained when utilizing the graphite as a template. Nitrogen adsorption and desorption analysis (Figure 3e–g) indicates that the mesoporous CeO_2_ hollow spheres, nanotubes, and nanosheets have a high surface area of 142 m^2^/g, 120 m^2^/g, 115 m^2^/g and uniform pore size of 3.3 nm, 3.4 nm, 3.4 nm, respectively. All the samples exhibit distinct mesoporous structures with different morphologies. This molten salt assisted assembly method greatly enriches the morphology of mesoporous metal oxides and achieves high crystallization while retaining the mesoporous channels, opening up the accessibility for synthesizing functional mesoporous metal oxides with diverse structures.

The mesoporous structure of mCeO_2_ provides a high surface area and transportation channel, and is particularly suitable for guest loading, confining, and functionalization. Cu nanoclusters possess excellent antibacterial properties and superior catalytic performance, while the Cu nanocluster is not stable in applications. Herein, the designed mCeO_2_ microspheres serve as a carrier for further modifying Cu species. Due to the homogeneous mesopores, the surface of mCeO_2_ microspheres can effectively adsorb and disperse Cu ions by postimpregnation. TEM images show the obtained Cu loaded mCeO_2_ (denoted as Cu-mCeO_2_) maintains the mesoporous structure well, and Cu elements are uniformly distributed in the mesopores over mCeO_2_ (Figure S15). BET analysis (Figure S16) indicates the obtained Cu-mCeO_2_ maintains well a high surface area of 132 m^2^/g and a narrow pore size distribution. The XRD patterns (Figure S17a) of Cu-mCeO_2_ show only the typical diffraction lines of CeO_2_ without other lines associated with Cu compounds as the Cu loading amount increased from 1.0 wt %, 2.0 wt %, to 4.0 wt % (feeding amount). The ICP-OES analysis (Figure S17b) reveals the actual Cu content is 0.87, 1.87, and 3.5 wt %, in accordance with the feeding amount. XPS spectra further verify the presence of Cu (Figure S17c–f), and the total spectrum shows that the peaks of Cu 2p on the surface gradually appear as the amount of doped Cu gradually increases. The concentration of vacancies and Ce^3+^ in mCeO_2_ showed a certain amount of decrease, which may be due to the reduction of Ce^3+^ for Cu^2+^ at a high temperature in the Ar atmosphere.

The antibacterial activity of commercial CeO_2_, mesoporous CeO_2_, and Cu-mCeO_2_ for Gram-positive (*Staphylococcus aureus*) and Gram-negative (*Escherichia coli*) strains was investigated. The positively charged Cu-cCeO_2_ and Cu-mCeO_2_ interacted with negatively charged bacterial strains, thereby inducing electrostatic attraction at the interface and triggering cell wall disruption. The disrupted cell wall allows the nanomaterials to enter the cellular compartment and thus generate reactive oxygen species.^39^ At the same time, their entry into the cellular compartment alters DNA and protein production and electron chain function, thereby preventing the entry of nutrients into the cell and promoting cellular inactivation.^40^

**Figure 4:**
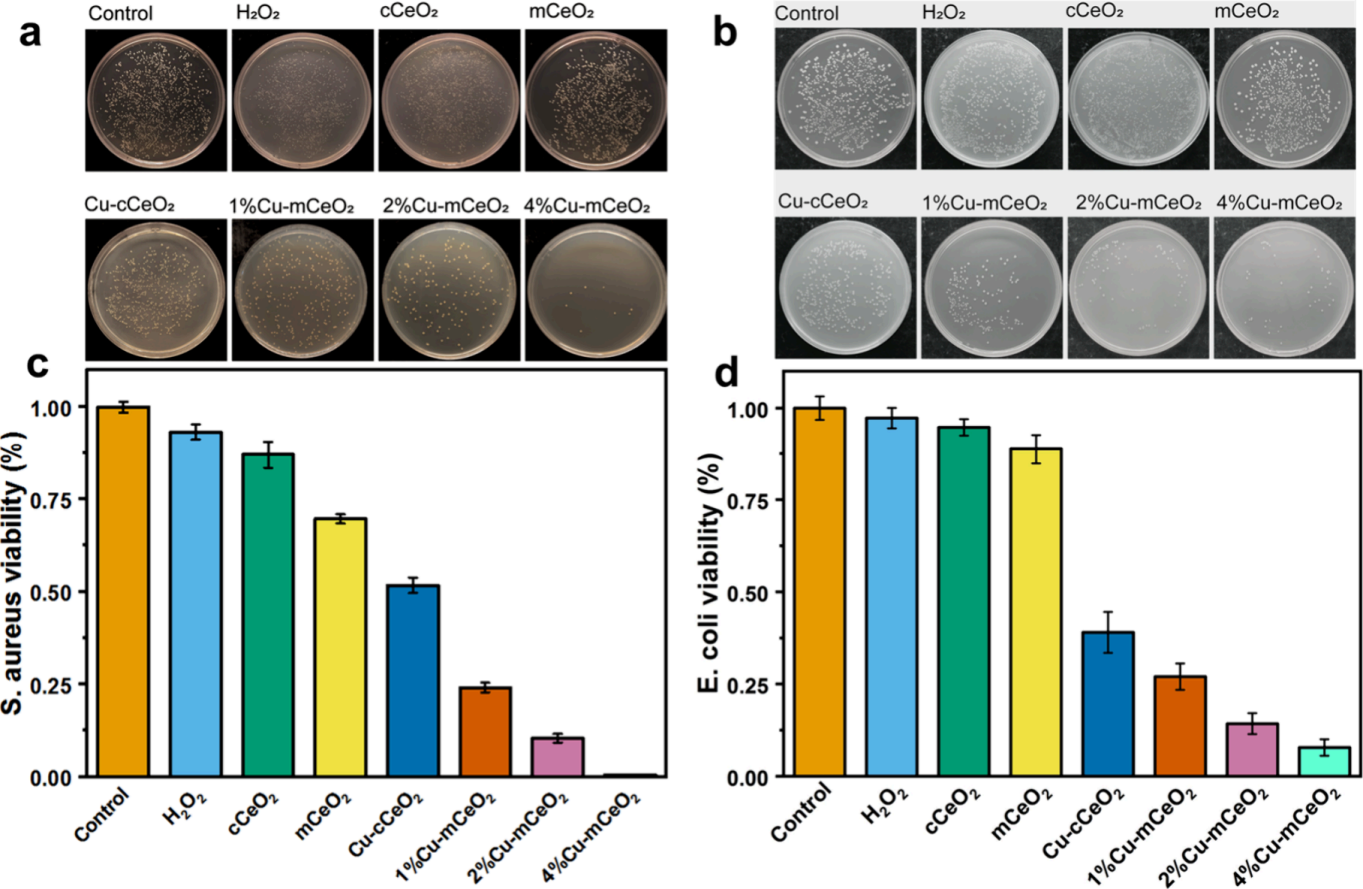
(a, b) Optical photographs of antibacterial activity by Cu-mCeO_2_ against *S. aureus* and *E. coli*; (c, d) graphs of viability of *E. coli* and *S. aureus* for Cu-mCeO_2_.

A comparison of the antibacterial activity of various CeO_2_ against the inhibition zone of bacterial strains is shown in Figure 4. The Cu-mCeO_2_ microspheres show significant antimicrobial activity both against *S. aureus* and *E. coli*, much higher antibacterial activity than that of commercial CeO_2_ with or without Cu doping. And the higher the concentration of Cu, the better antibacterial activity of Cu-mCeO_2_ with *S. aureus* and *E. coli*, indicating that Cu ions are the main active site for antibacterial activity. The 4% Cu-mCeO_2_ exhibits the highest antibacterial effect, which is far better than the commercial CeO_2_. It indicates that the large surface area and open pore channels facilitate the increase of reaction area and the good dispersion of Cu sites, which accelerates the reaction process and effectively hinders the growth of bacteria.

**Figure 5:**
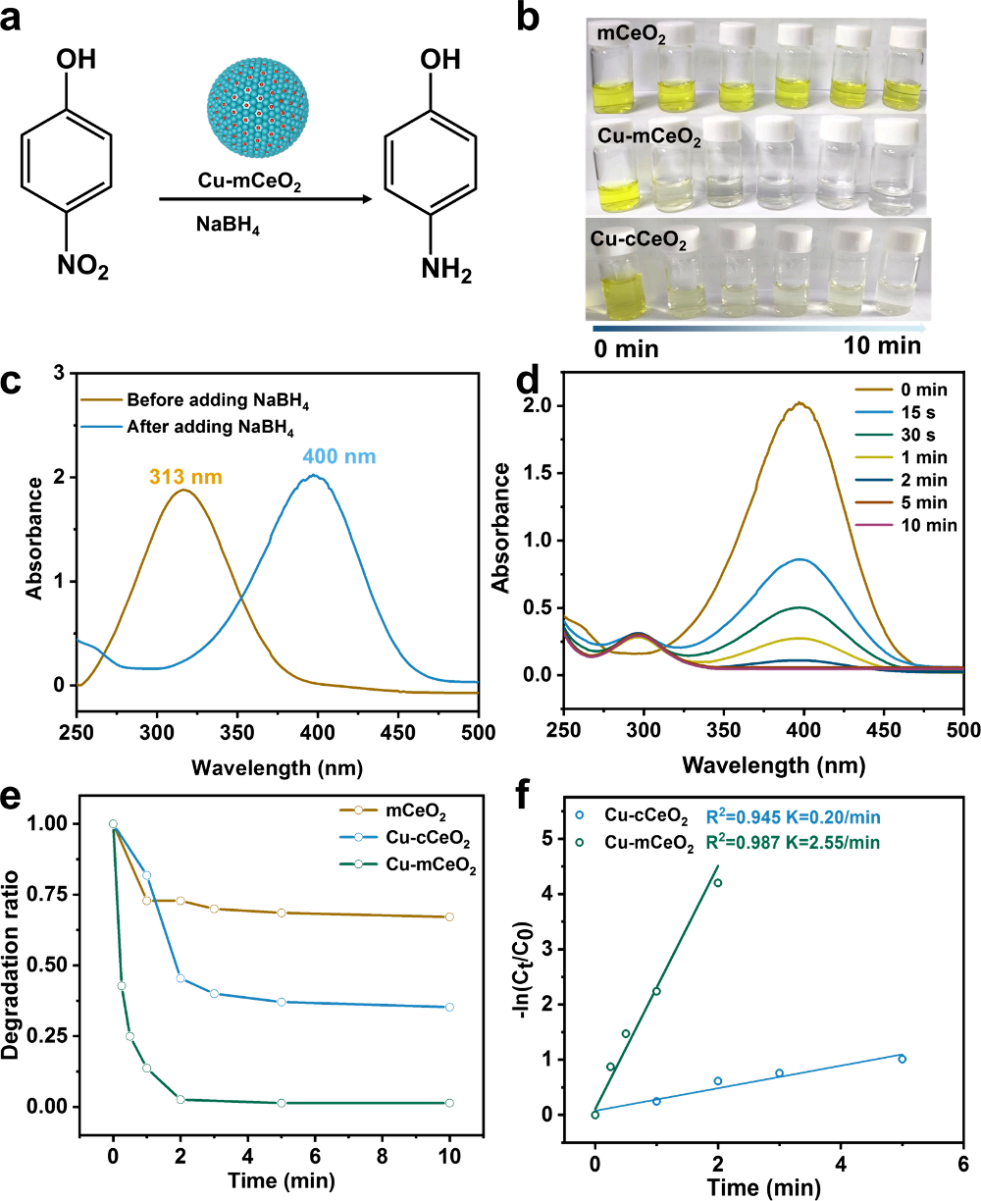
(a) Cu-mCeO_2_ catalyzed hydrogenation of 4-NP; (b) color change of the solution during reaction; (c) UV–vis absorption spectrogram with and without NaBH_4_; (d) UV–vis absorption spectrogram at various time ranges for Cu-mCeO_2_; (e) degradation rate and (f) reaction rate diagram of the catalysis process.

Owing to its high chemical stability, large surface area, and numerous mesoporous pores, mCeO_2_ is particularly suitable as a heterogeneous catalyst carrier. With well-dispersed Cu sites confined in the mesopores, the catalytic activity of Cu-mCeO_2_ for the reduction of 4-nitrophenol (4-NP) was investigated. The upgrading of 4-nitrophenol (4-NP) to the high-value 4-aminophenol (4-AP) by mild heterogeneous hydrogenation is of significance in industrial synthesis, since 4-AP is an important chemical intermediate in the synthesis of pharmaceuticals, dyes, corrosion inhibitors, and agrochemical and imaging agents. Ultraviolet–visible (UV–vis) spectrophotometry was utilized to detect the catalytic performance of Cu-mCeO_2_ catalyst (Figure 5a,b). When NaBH_4_ was added, the UV–vis absorption peak of 4-NP shifted from 313 to 400 nm as a result of the deprotonation reaction of 4-NP (Figure 5c). However, the UV–visible absorbance no longer changes over time, proving that the hydrogenation process does not happen in the absence of catalysts. Following the injection of Cu-mCeO_2_, 4-NP is rapidly consumed, and the absorption peak (400 nm) decreases within 2 min until it completely disappears (Figure 5d,e). On the contrary, the degradation efficiency of mCeO_2_ and Cu-cCeO_2_ is significantly lower than that of Cu-mCeO_2_ (Figure 5e, S18a,b). A good linear correlation is established by evaluating the relationship between ln(*C*_t_/*C*_0_) (where *C*_t_ and *C*_0_ reflect the amount of 4-NP at *t* and 0 min, respectively, and correspond to the absorbance at *A*_0_). The slopes of Cu-mCeO_2_ and Cu-cCeO_2_ were 2.55 min^–1^ and 0.57 min^–1^, respectively (Figure 5f). It suggests that the Cu-mCeO_2_ catalysts can significantly accelerate the catalytic reaction process due to the high catalytic activity, large reactive area, and nanoconfined and well-dispersed active sites. Table S1 summarizes the comparison of the catalytic activity with reported catalysts, and the Cu-mCeO_2_ shows a superior catalytic activity to the reported nonprecious catalysts and even a comparable performance with the precious metal catalysts.

Figure S18c shows the cycling performance of Cu-mCeO_2_, which is maintained above 94% conversion of the 4-nitrophenol after 5 cycles, indicating superior durability. The catalysts show high catalytic performance for the reduction of other nitrophenols (Figure S19). The TEM images of the recovered Cu-mCeO_2_ catalyst reveal the excellent structural stability, and the XRD pattern confirms that the crystal structure of mCeO_2_ is well maintained. The ICP-MS results reveal that the concentrations of Cu and Ce ions in the catalytic solution were negligible (10 ppb for Cu and 38 ppb for Ce), indicating the excellent stability of the confined Cu species in the mCeO_2_ (Figure S20).

## Conclusions

In summary, a facile and versatile molten-salt assisted assembly method is developed to synthesize mesoporous metal oxide with various components and nanostructures. On one hand, the molten salt endows desolvation of the precursor to form bare metal ions and enhances the coordination interaction between metal ion and ether oxygen atom, thus boosting their assembly into mesostructures. On the other hand, the molten salt provides a liquid crystallization microenvironment, which allows the in situ crystallization of grains and inhibits their further growth into large crystals, thus ensuring the high crystallization as well as stable mesoporous structures. Various mesoporous metal oxides (CeO_2_, ZrO_2_, SnO_2_, Li_2_TiO_3_) and diverse nanostructures (microspheres, hollow spheres, nanotubes, and nanosheets) were successfully fabricated through this facile method. The obtained mesoporous CeO_2_ microspheres with a surface area of 152.2 m^2^/g and pore size of 3.0 nm served as a carrier for loading Cu species, and Cu-mCeO_2_ exhibits excellent antimicrobial performance as well as superior heterogeneous catalytic activity and high stability for hydrogenation of nitrophenols. The developed molten-salt assisted assembly method opens up a new avenue for design synthesis of functional and highly crystallized mesoporous metal oxides.

## Supporting Information Available

Detailed description of synthesis, antibacterial assay, catalysis, and materials characterization including XRD, TG, IR, TEM, SEM, N_2_ adsorption and desorption isotherms, etc. (PDF)
