# Supplementary material for: A Novel Molten Salt Mediated Synthesis of Mesoporous Metal Oxides with High Crystallization
Source: ACS Cent Sci. 2024 Feb 26;10(3):676–83. doi: 10.1021/acscentsci.3c01462 (PMC10979477; doi:10.1021/acscentsci.3c01462)
Supplement: Supplementary file 1 — oc3c01462_si_001.pdf [file oc3c01462_si_001.pdf]

A Novel Molten Salt Mediated Synthesis of Mesoporous Metal Oxides with High Crystallization

Dongsheng Ma[1], Hanpeng Lu[2], Yu Zhou[1], Shuaihu Jiang[1], Duan Wang[2], Qin Yue[1]*

[1] Institute of Fundamental and Frontier Sciences, University of Electronic Science and Technology of China, Chengdu 610054, China

[2] Orthopedic Research Institution, Department of Orthopedics, West China Hospital, Sichuan University, Chengdu 610041, China.

* Corresponding authors. Email: qinyue@uestc.edu.cn

Experimental Section:

Chemicals. $Ce(SO_4)_2 \cdot 4H_2O$, KNO_3, $LiNO_3$, $Zr(SO_4)_2 \cdot 4H_2O$, $SnCl_2 \cdot 2H_2O$, $Ti(SO_4)_2 \cdot 6H_2O$, $Co(ac)_2 \cdot 4H_2O$ and $Ni(ac)_2 \cdot 4H_2O$ are purchased from Aladdin. Pluronic F127 and H_2O_2 (30%) are purchased from Sigma-Aldrich. HNO_3 (65 wt%-68wt%) was purchased from Adamas.

Synthesis of $mCeO_2$ microspheres:

Typically, F127 (0.4 g), and $Ce(SO_4)_2 \cdot 4H_2O$ (1 mmol) were added to the mortar and grinding for 3 minutes. Then, 10 mmol nitrate (molar ratio KNO_3: $LiNO_3$=0.57:0.43) was added to the above mortar and grinding for 3 minutes. The mixture is transferred to the crucible and placed into the muffle furnace for calcination. The heating up procedure is as follows: first, the temperature rises from room temperature to 160 ℃ at 2 ℃/min, keeps at 160 ℃ for three hours and then rises to 400 ℃ at 2 ℃/min with preservation for three hours. Finally muffle furnace naturally cools to room temperature. Then, the salt is removed by centrifugation and washing with deionized water for more than four times, and finally the $mCeO_2$ microspheres are dried in an oven at 70 ℃.

Synthesis of $mCeO_2$ hollow spheres:

The polydopamine microspheres, were calcined in N_2 at 350 ℃ for 3 h in order to obtain a pre-carbonisation. The obtained carbon microspheres (0.1 g), F127(0.3 g), 1 mmol $Ce(SO_4)_2 \cdot 4H_2O$ were added to the mortar and grinding for 3 minutes. Then, 10 mmol nitrate (molar ratio KNO_3: $LiNO_3$=0.57:0.43) were added to the above mortar and grinding for 3 minutes, then the mixture is transferred to the crucible and placed into the muffle furnace for calcination. The temperature rise procedure is as follows: first, the temperature rises from room temperature to 160 ℃ at 2 ℃/min, keeps at 160 ℃ for three hours and then rises to 400 ℃ at 2 ℃/min with preservation for three hours. Finally muffle furnace naturally cools to room temperature. Finally, the $mCeO_2$ hollow spheres were dried in oven at 70 ℃ after removing the salt by centrifugation and washing with deionized water more than four times.

Synthesis of $mCeO_2$ nanotubes:

Soak carbon nanofibers in hydrogen peroxide for pretreatment (sCNF). 0.3 g of F127, 0.1 g of sCNF and 1 mmol $Ce(SO_4)_2 \cdot 4H_2O$ were added to the mortar and grinding for 3 minutes, 10 mmol nitrate (molar ratio KNO_3: $LiNO_3$=0.57:0.43) were added to the above mortar and grinding for 3 minutes, then the mixture is transferred to the crucible and placed into the muffle furnace for calcination. The temperature rise procedure is as follows: first, the temperature rises from room temperature to 160 ℃ at 2 ℃/min, keep at 160 ℃ for three hours and then rises to 400 ℃ at 2 ℃/min with preservation for three hours. Finally muffle furnace naturally cools to room temperature. Finally, the hollow $mCeO_2$ nanotubes were dried in oven at 70 ℃ after removing the salt by centrifugation and washing with deionized water more than four times.

Synthesis of $mCeO_2$ nanosheets:

Firstly, the graphite powder is immersed into dilute nitric acid (40%) for pretreatment (sGP). 0.3 g of F127, 0.1 g of sGP and 1 mmol $Ce(SO_4)_2 \cdot 4H_2O$ were added to the mortar and grinding for 3

minutes, then 10 mmol nitrate (molar ratio KNO_3: $LiNO_3$=0.57:0.43) were added to the above mortar and grinding for 3 minutes, then the mixture is transferred to the crucible and placed into the muffle furnace for calcination. The temperature rise procedure is as follows: first, the temperature rises from room temperature to 160 °C at 2 °C/min, keep at 160 °C for three hours and then rises to 400 °C at 2 °C/min with preservation for three hours. Finally muffle furnace naturally cools to room temperature. Finally, the mCeO_2 nanosheets were dried in oven at 70 ℃ after removing the salt by centrifugation and washing with deionized water more than four times.

Synthesis of mesoporous ZrO_2, SnO_2, Li_2TiO_3 and (CeZrTiSnCoNi)O_x:

The synthesis of mesoporous ZrO_2, SnO_2 and Li_2TiO_3 is similar with that of mCeO_2 microspheres except the precursor $Ce(SO_4)_2 \cdot 4H_2O$ is replaced by $Zr(SO_4)_2 \cdot 4H_2O$, $SnCl_2 \cdot 2H_2O$ and $Ti(SO_4)_2 \cdot 6H_2O$, respectively. A similar synthesis procedure applies to high-entropy oxides (CeZrTiSnCoNi)O_x accompanied by equal amounts of $Ce(SO_4)_2 \cdot 4H_2O$, $Zr(SO_4)_2 \cdot 4H_2O$, $Ti(SO_4)_2 \cdot 6H_2O$, $SnCl_2 \cdot 2H_2O$,$Co(ac)_2 \cdot 4H_2O$ and $Ni(ac)_2 \cdot 4H_2O$ as precursors.

Characterization:

The absorbance of the material is tested by Fourier Transform infrared spectroscopy (FTIR), ranging from 400-4000 cm^{-1} (Nicolet Nexus 470). The X-ray diffraction (XRD) of the powder was measured in the ARL EQUINOX 1000 with Cu Kα (λ = 0.154 nm) in the range of 10-80 °. The N_2 adsorption/desorption curve is characterised on ASAP2460 after vacuum degassing at 180 ° for 8h. SEM (Zeiss Gemini 300), TEM (JEM-1400FLASH 120kV), HRTEM and EDS-mapping (Tecnai G2 F20 S-TWIN, FEI 200kV) are used to characterise the morphology of the samples. X-ray photoelectron spectrometer (XPS, Thermo Scientific K-Alpha 250Xi) equipped with Al-Kα source and hemispherical chemical analyser. ICP-OES (Agilent 720ES) was used to measure metal concentrations (ppm level). Thermogravimetric analysis (TG 209F3) curve by heating of sample in air from room temperature to 800 ℃ (rate: 5 ℃/min). The Raman (Horiba LabRAM HR Evolution) test is performed with a 540 nm laser in the range of 50 to 4000 cm^{-1}.

Antibacterial assay

Two bacterial strains, Escherichia coli (E.coli, ATCC 25922) and Staphylococcus aureus (S.aureus, ATCC 25923) were tested for antibacterial properties of the materials. Specifically, the materials were treated with bacterial suspension (500 μL, 1×105 CFU/mL) into EP tubes to achieve a concentration of 100 ug/ml of the materials, and then hydrogen peroxide was added to the experimental groups to achieve a concentration of 1mM. All groups were incubated at 37 ℃ for 300 minutes. Afterwards, bacterial suspension was collected from each EP tube to LB agar plate at 37 °C for 12 hours to obtain bacterial colonies.

Catalytic reduction of nitrophenols

In general, 15 mg of catalyst was added to 50 mL of freshly prepared aqueous 4-NP solution (0.03 mmol), followed by the addition of 0.05 g of $NaBH_4$, with constant stirring at room

temperature. At regular intervals, 2.5 mL of the reaction solution was withdrawn and filtered through a 0.2 μm membrane, and the absorbance was measured using a UV-Vis spectrophotometer. In the cyclic test, after each reaction, the catalyst was washed by centrifugation and then dried in a vacuum oven before the next reaction.

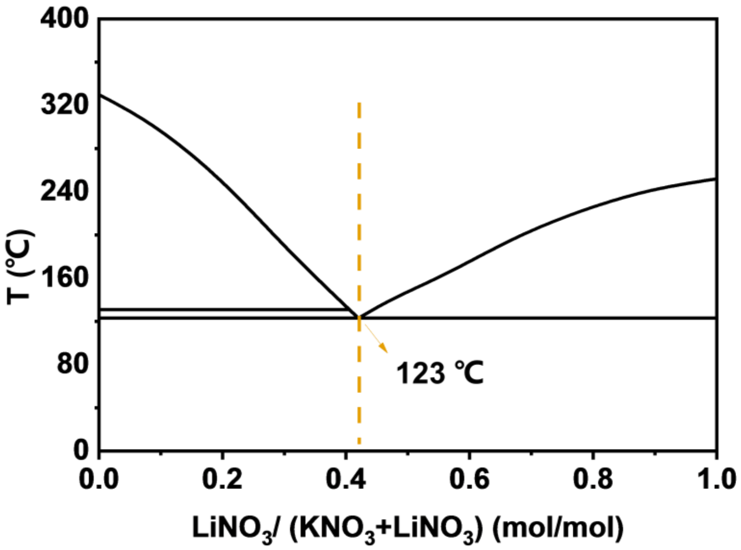

Figure S1. Eutectic phase diagram of $LiNO_3$-KNO_3.

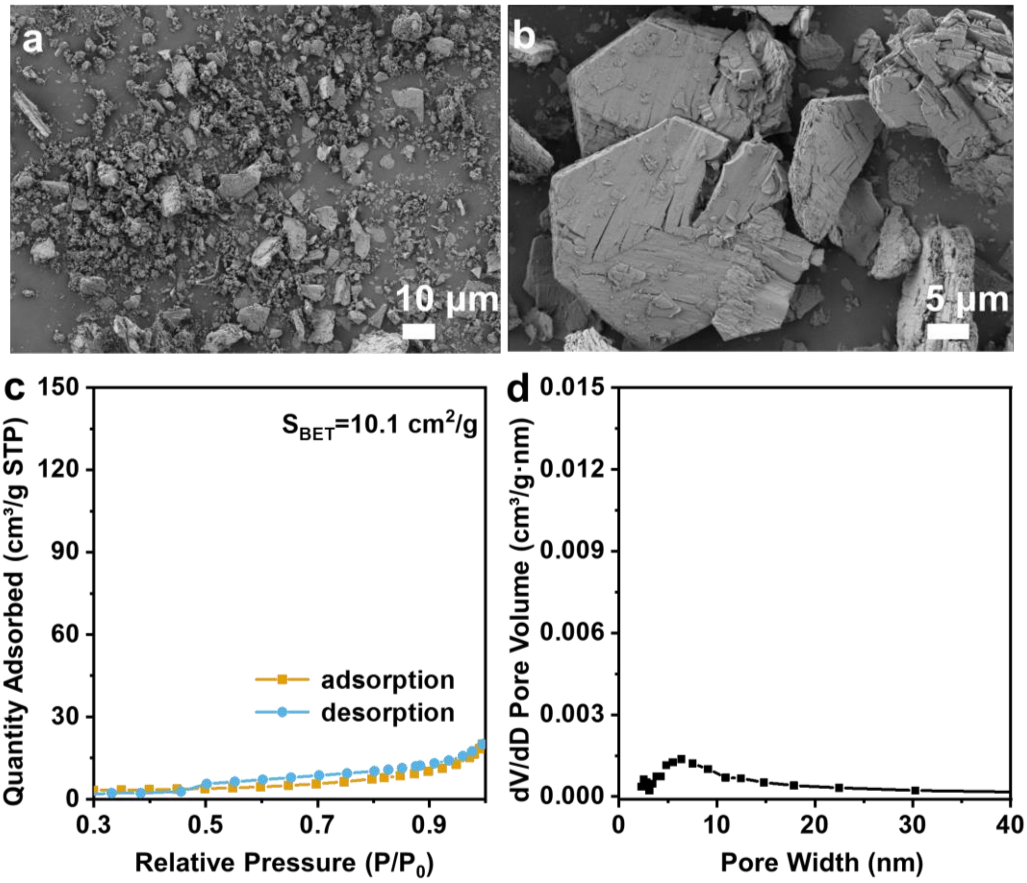

Figure S2. The characterizations of the commercial CeO_2. (a)SEM images; (b) Nitrogen adsorption and desorption isotherms; (c) pore size distribution curve.

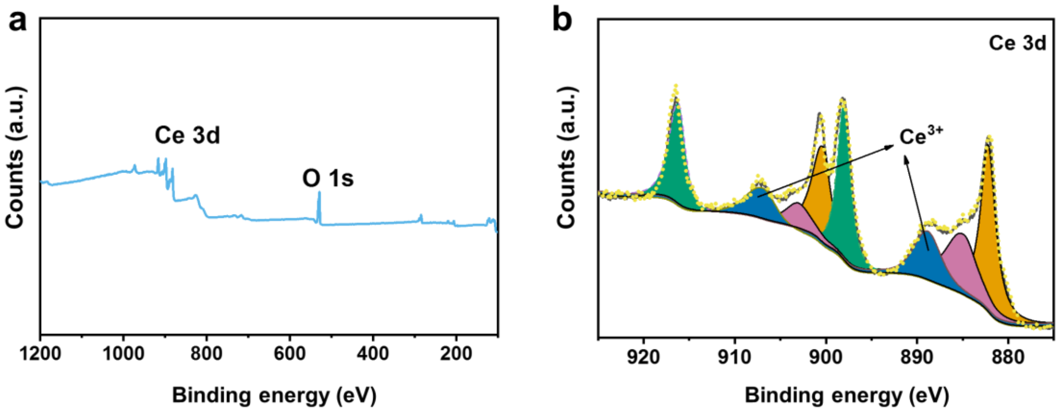

Figure S3. (a) XPS full spectrum and (b) Ce 3d spectrum for the $mCeO_2$ microsphere.

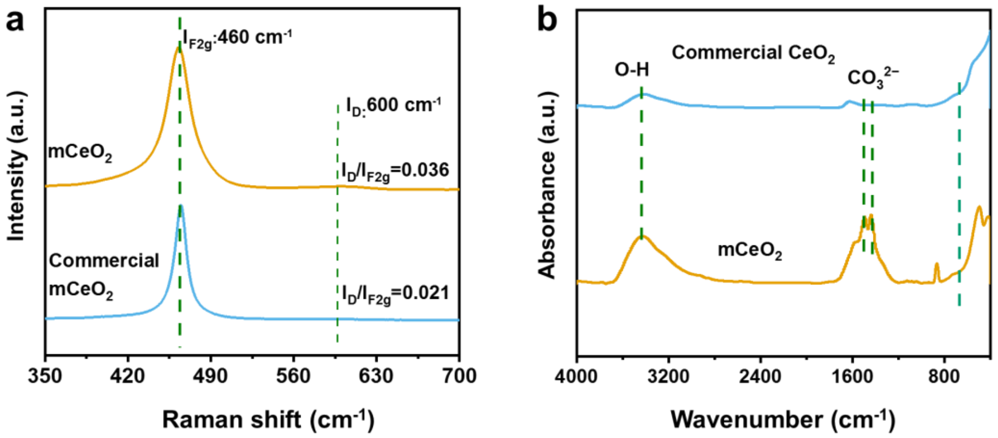

Figure S4. (a) FTIR spectra and (b) Raman spectra of $mCeO_2$ and commercial CeO_2, (c) thermogravimetric curves of $mCeO_2$ microspheres.

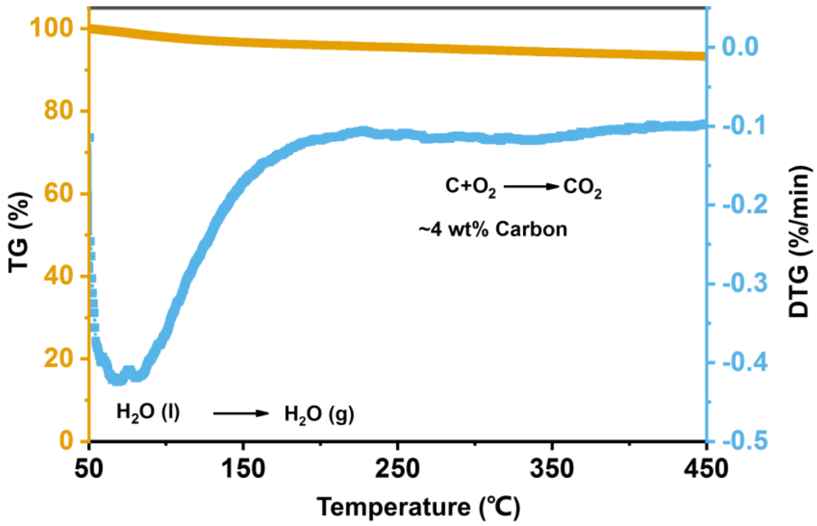

Figure S5. Thermogravimetric curves of mCeO_2 microspheres.

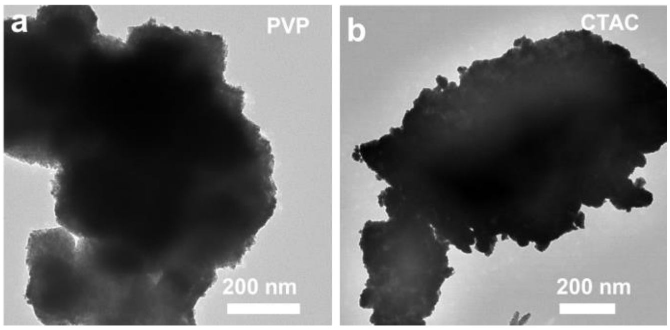

Figure S6. TEM images of the CeO_2 synthesized by replacing F127 with (a) PVP and (b) CTAC.

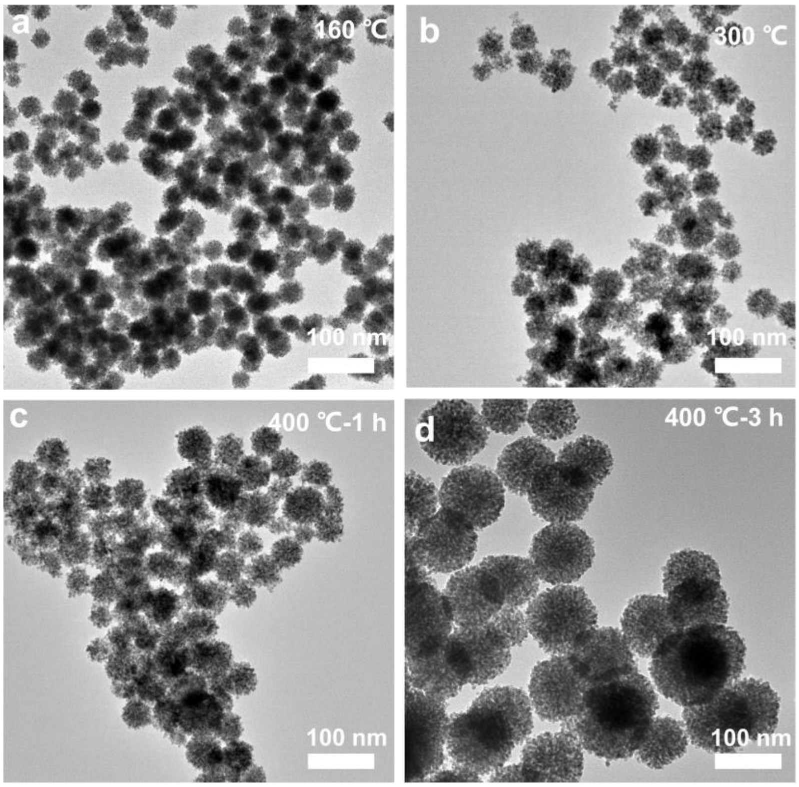

Figure S7. TEM images of $mCeO_2$ at different reaction temperatures and time. (a) 160 ℃; (b) 300 ℃; (c) 400 ℃ for 1 hour; (d) 400 ℃ for 3 hours.

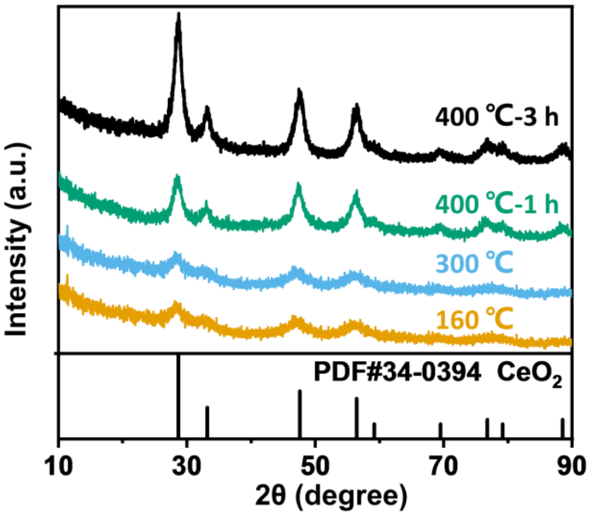

Figure S8. XRD patterns of $mCeO_2$ at different reaction temperatures and reaction time. (a) 160 ℃; (b) 300 ℃; (c) 400 ℃ for 1 hour; (d) 400 ℃ for 3 hours.

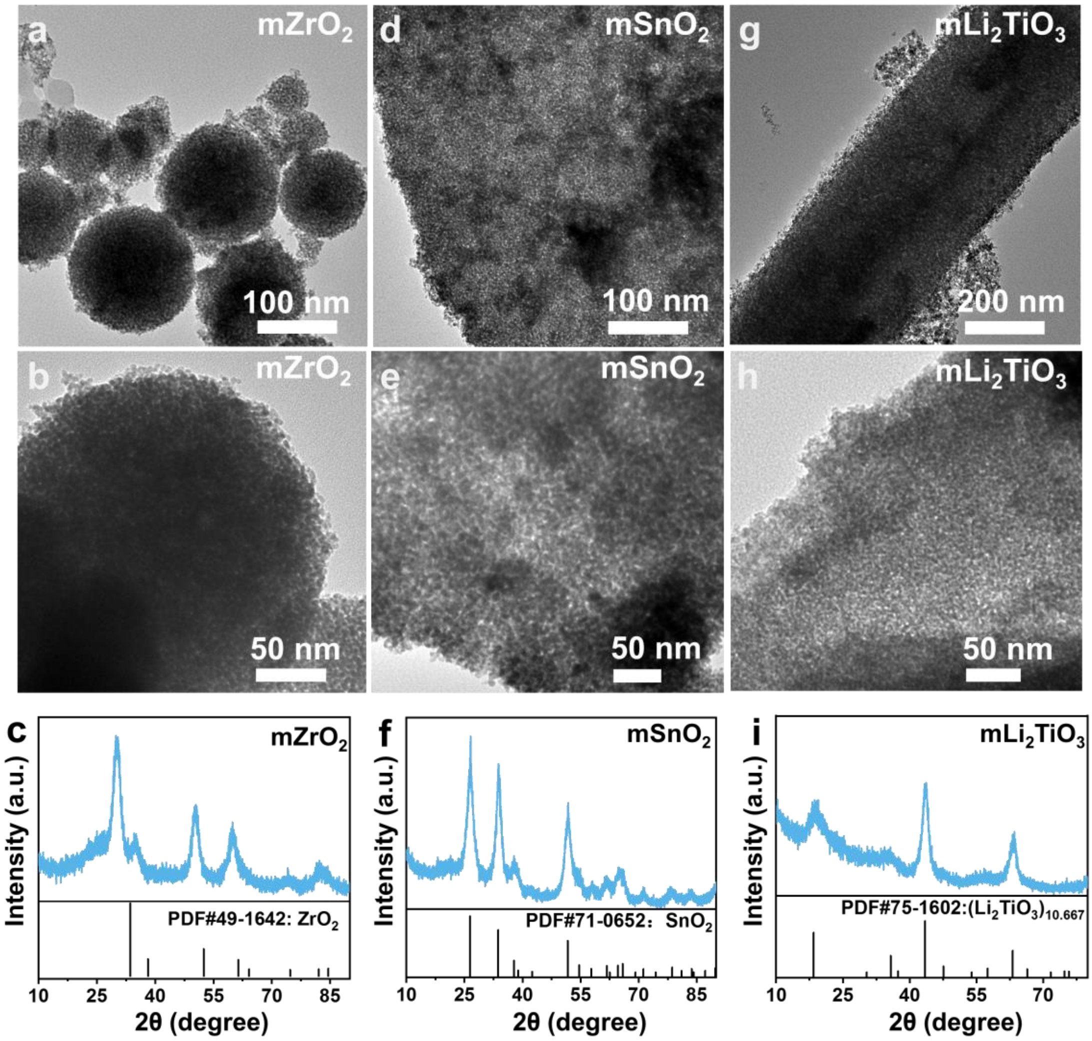

Figure S9. TEM images and XRD patterns of (a-c) mesoporous ZrO_2, (d-f) mesoporous SnO_2, and (g-i) mesoporous Li_2TiO_3.

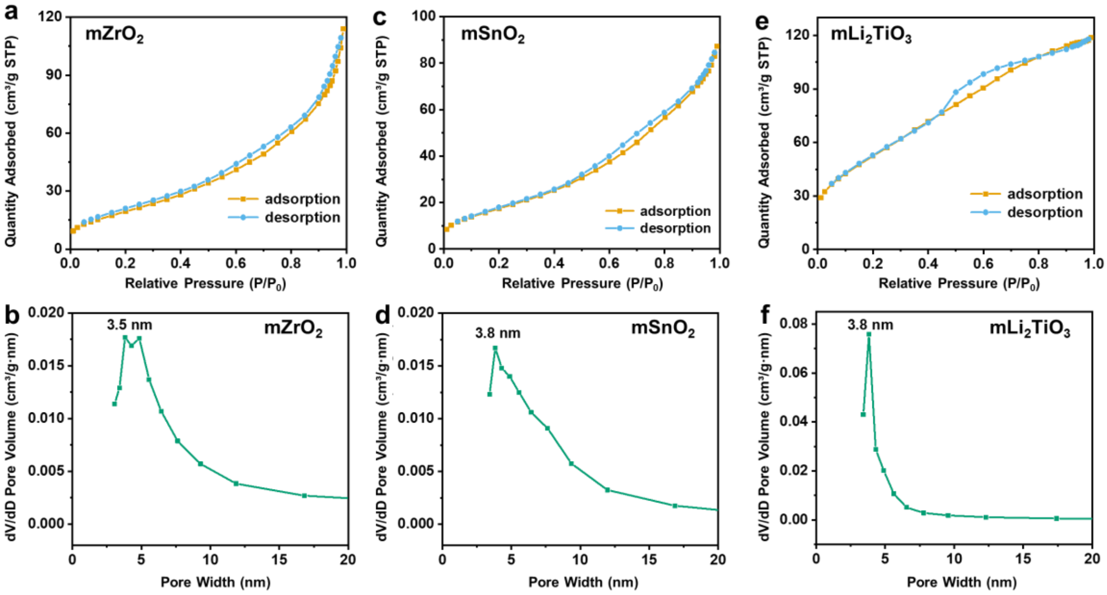

Figure S10. Nitrogen adsorption and desorption isotherm, and pore size distribution of (a-b) mesoporous ZrO_2, (c-d) mesoporous SnO_2, and (e-f) mesoporous Li_2TiO_3.

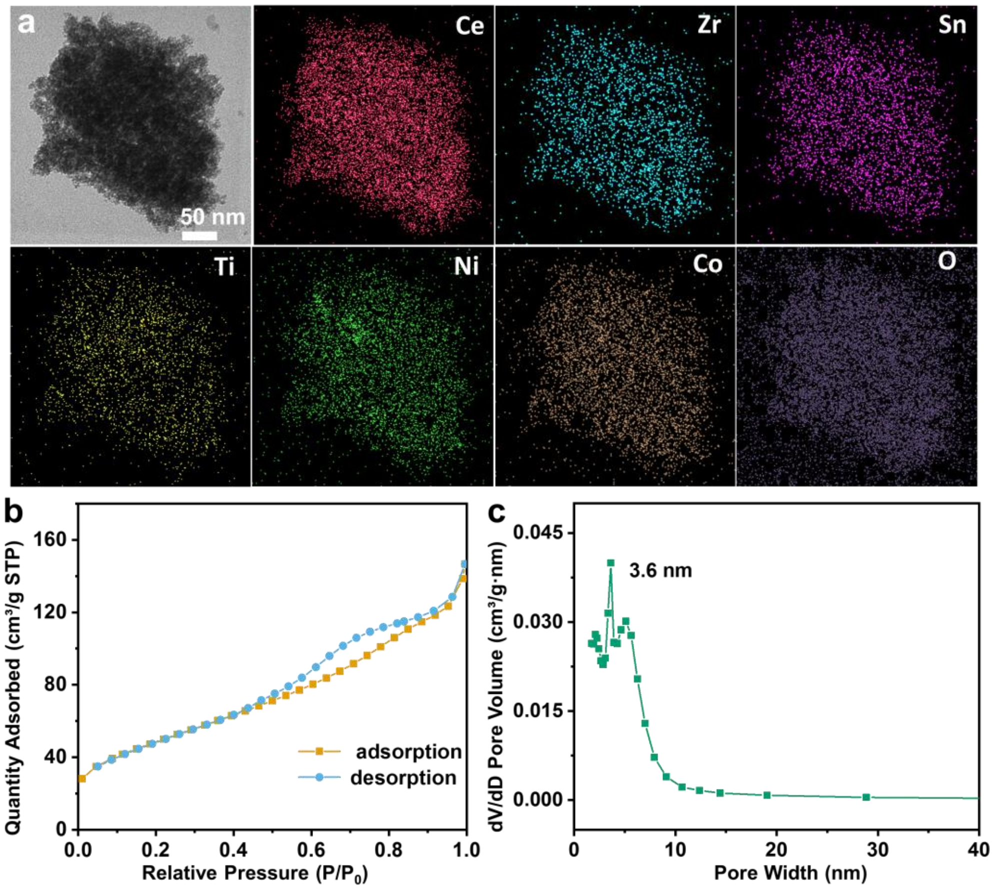

Figure S11. Characterization of mesoporous high-entropy metal oxides (CeZrTiSnCoNi)O_x. (a) TEM images and EDS mapping; (b) nitrogen adsorption and desorption isotherms; (c) pore size distribution curve.

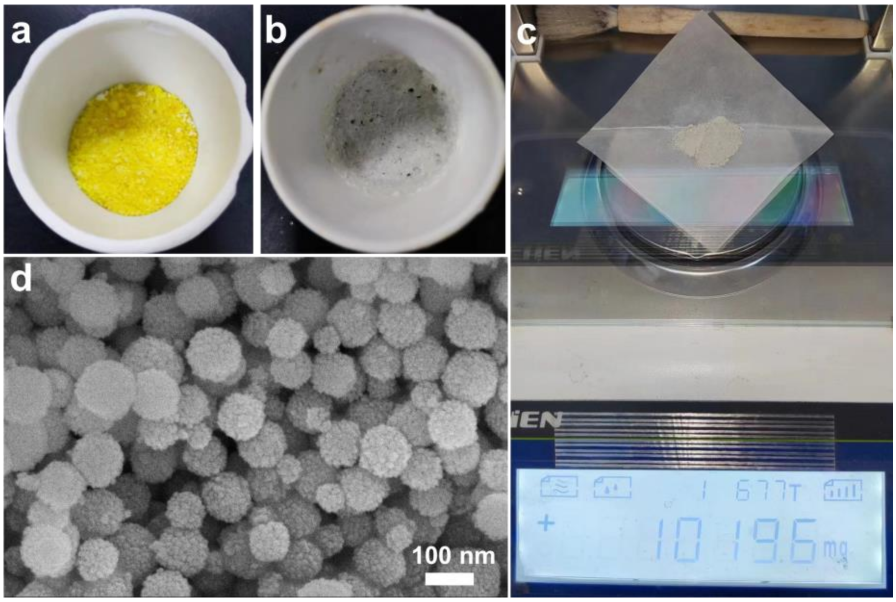

Figure S12. The optical photographs. (a) precursor after milling; (b) as-made sample after reaction; (c) the obtained $mCeO_2$ with a g-scale yield; (d) SEM image of the $mCeO_2$.

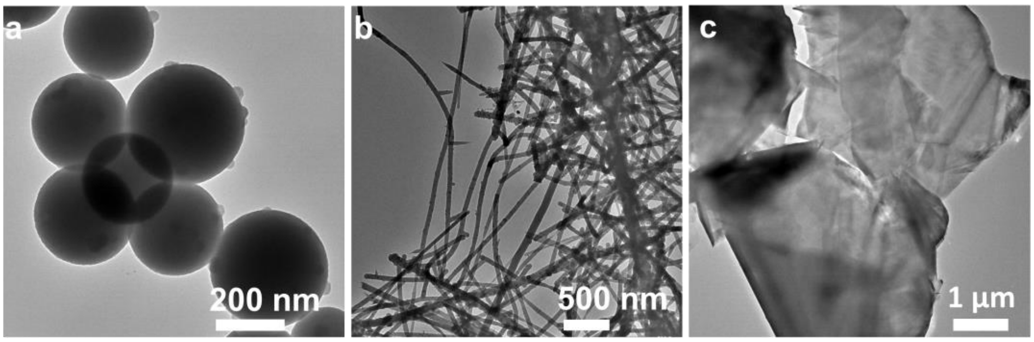

Figure S13. TEM images of different carbon template. (a) carbon microsphere; (b) carbon nanofibre; (c) carbon nanosheet.

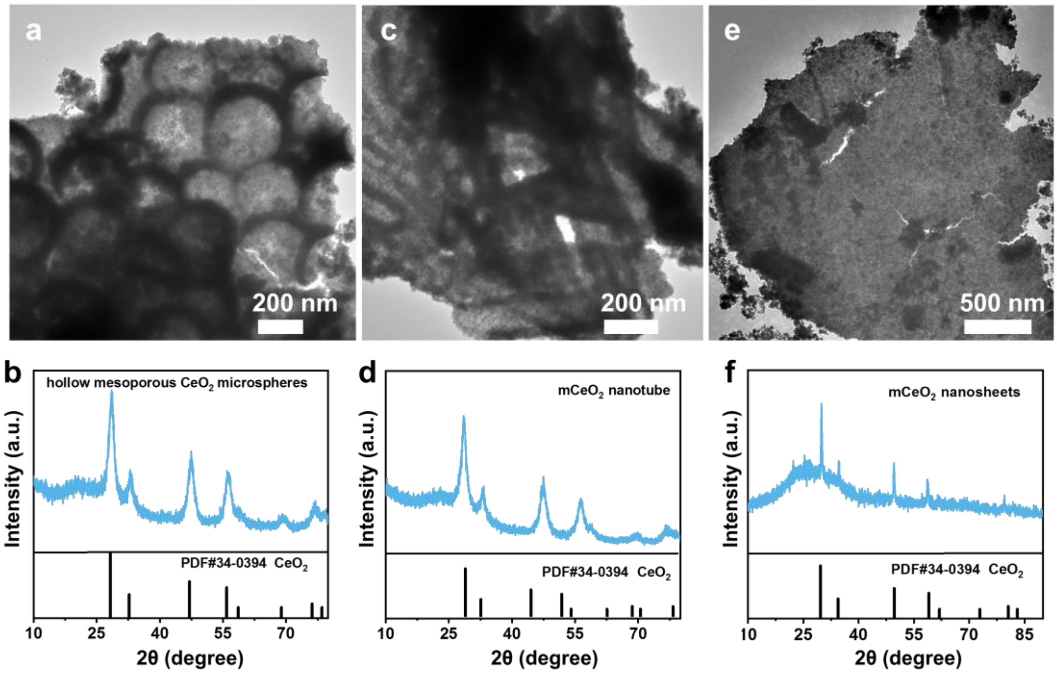

Figure S14. TEM images (a, c, e) and XRD patterns (b, d, f) of $mCeO_2$ with various morphology. (a, b) $mCeO_2$ hollow spheres; (c, d) $mCeO_2$ nanotubes; (e, f) $mCeO_2$ nanosheets.

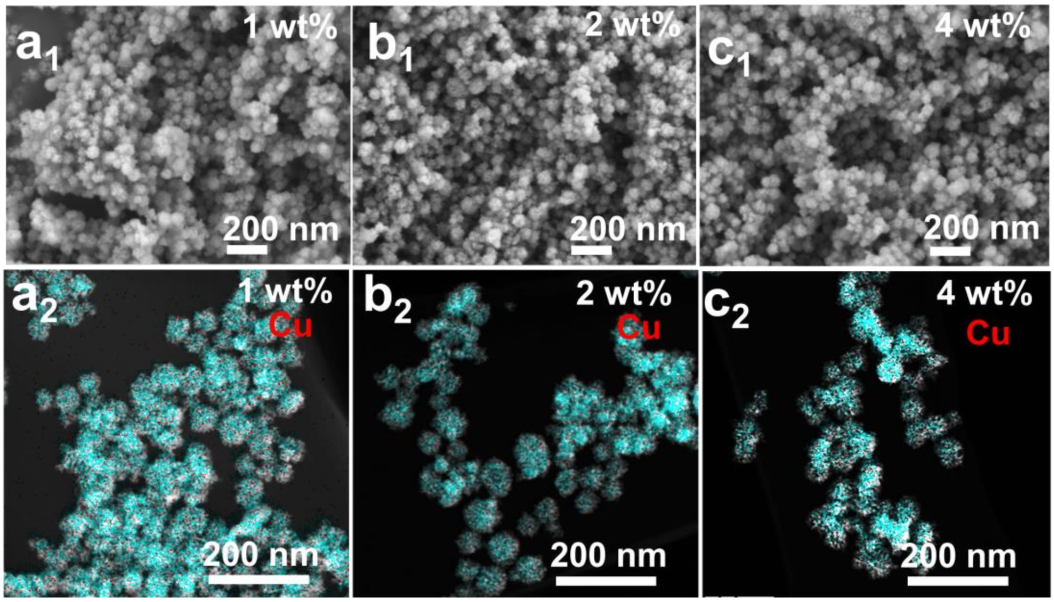

Figure S15. SEM and Mapping images of $mCeO_2$ with different Cu loadings. (a) 1wt%Cu-$mCeO_2$; (b) 2wt%Cu-$mCeO_2$; (c) 4wt%Cu-$mCeO_2$.

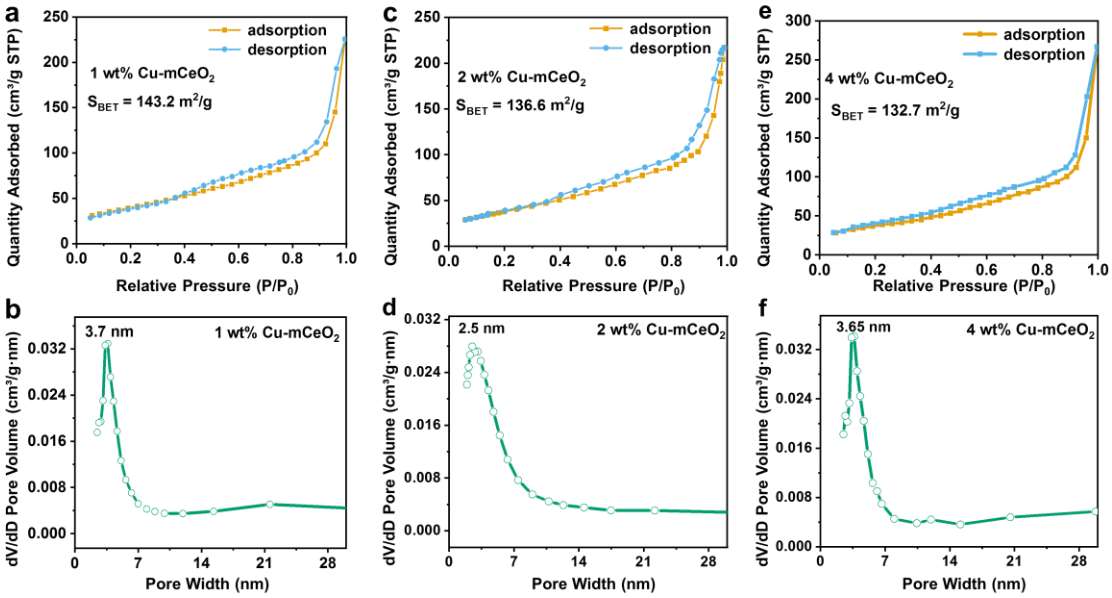

Figure S16. Nitrogen adsorption and desorption isotherm, and pore size distribution. (a-b) 1wt% Cu-mCeO_2; (c-d) 2wt% Cu-mCeO_2; (e-f) 4wt% Cu-mCeO_2.

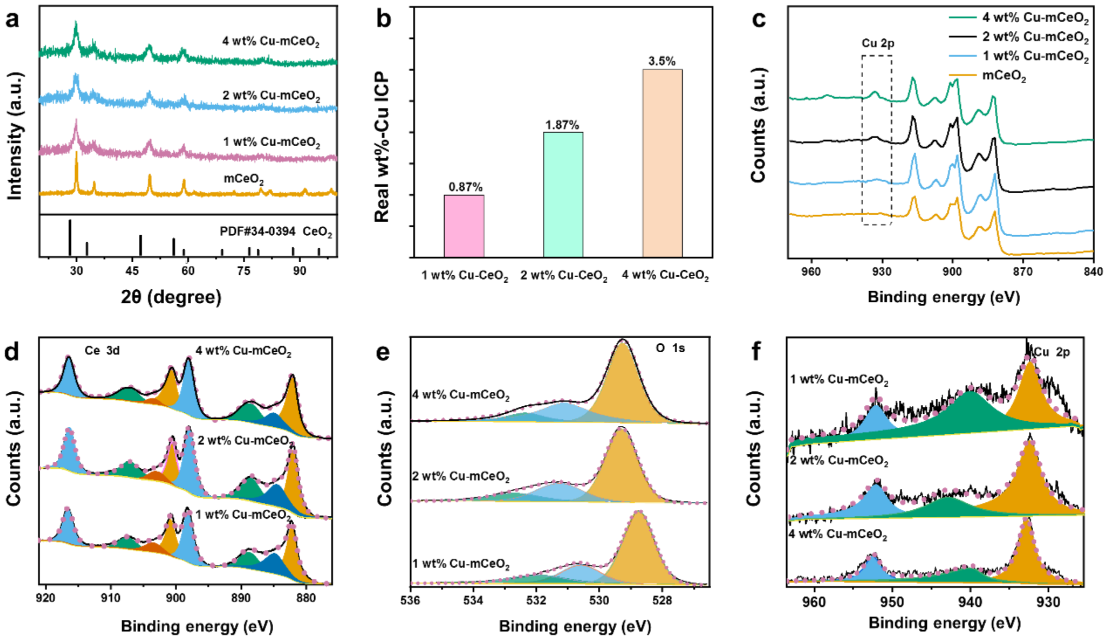

Figure S17. The characterizations of $mCeO_2$ with different Cu loadings. (a) XRD patterns; (b) the content of Cu in Cu-$mCeO_2$ (the values were calculated based on ICP results); (c) XPS survey spectrum; (d-f) XPS spectra of Ce 3d, O 1s and Cu 2p.

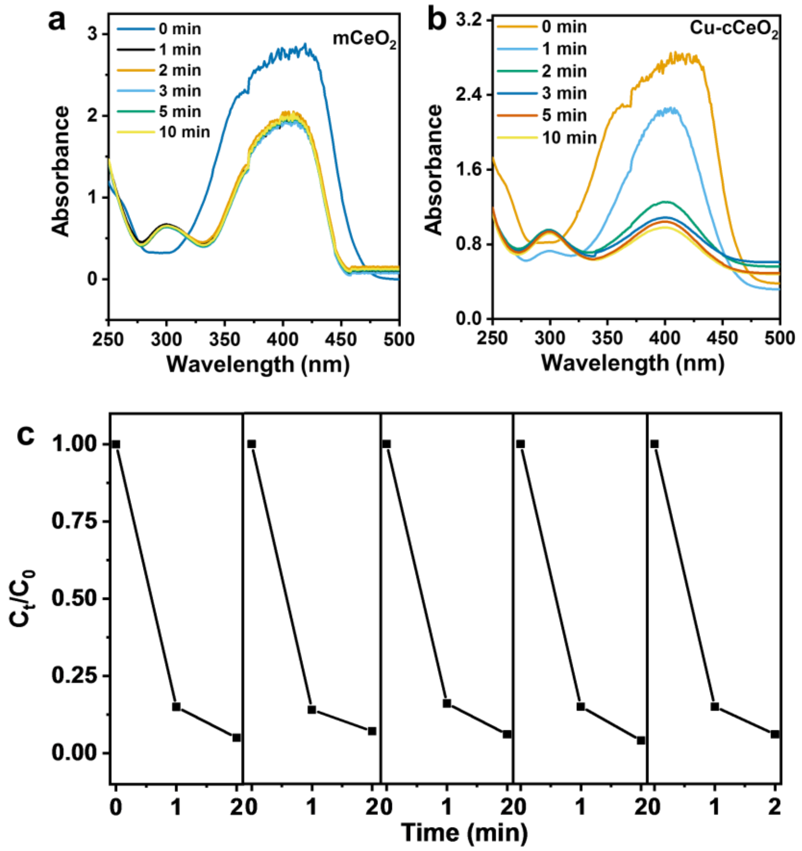

Figure S18. (a) UV-vis absorption spectrogram of the reaction solution at various time in the catalysis of mCeO_2 microspheres; (b) UV-vis absorption spectrogram of the reaction solution at various time in the catalysis of the Cu-cCeO_2; (c) Cu-mCeO_2 catalyses the conversion of 4-NP to 4-AP for 5 cycles.

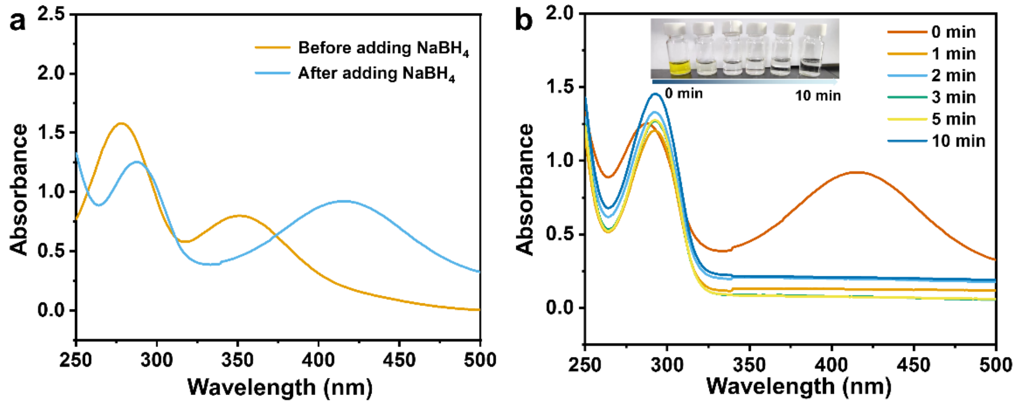

Figure S19. (a) UV-vis absorption spectrogram with and without $NaBH_4$ for o-Nitrophenol, (b) UV-vis absorption spectrogram of Cu-mCeO_2 at various time for o-Nitrophenol.

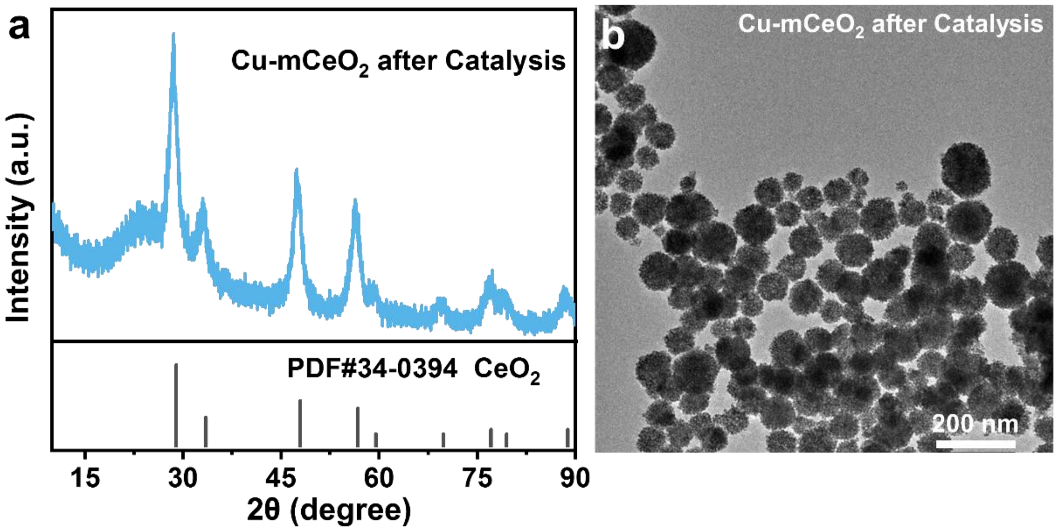

Figure S20. (a) XRD pattern and (b)TEM pattern of Cu-mCeO_2 after the cycling.

Table 1. Comparative performance of catalytic conversion of nitrophenols

Catalyst	Rate constant (min^{-1})	Rate constant unit mass (min^{-1} g^{-1})	Ref
Cu@mCeO_2	2.55	170	This work
Ni-Ca-Al_2O_3	0.171	17.1	[1]
SSBC-800	0.48	48	[2]
PCCN	0.549	54.9	[3]
Co/EAtp@C	0.69	69	[4]
Ni/NiO-450	0.440	87.91	[5]
Cu-Mag	0.223	111.2	[6]
Au@ZIF-8	0.483	15.3	[7]
ACOS	0.122	48.7	[8]
Pd1@np-Ni/NiO	0.635	127	[9]
PtNi/SiO_2	0.77	154	[10]

Reference

[1] Feng, J.; Wang, Q.; Fan, D.; Ma, L.; Jiang, D.; Jiang, D.; Zhu, J. Nickel-based xerogel catalysts: Synthesis via fast sol-gel method and application in catalytic hydrogenation of p-nitrophenol to p-aminophenol. *Applied Surface Science* **2016**, *382*, 135-143.

[2] Ren, X.; Tang, L.; Wang, J.; Almatrafi, E.; Feng, H.; Tang, X.; Yu, J.; Yang, Y.; Li, X.; Zhou, C.; Zeng, Z.; Zeng, G. Highly efficient catalytic hydrogenation of nitrophenols by sewage sludge derived biochar. *Water Research* **2021**, *201*, 117360.

[3] Huang, T.; Fu, Y.; Peng, Q.; Yu, C.; Zhu, J.; Yu, A.; Wang, X. Catalytic hydrogenation of p-nitrophenol using a metal-free catalyst of porous crimped graphitic carbon nitride. *Applied Surface Science* **2019**, *480*, 888-895.

[4] Zhang, S.; Zhong, L.; Xu, Z.; Hu, J.; Tang, A.; Zuo, X. Mineral-modulated Co catalyst with enhanced adsorption and dissociation of BH_4^- for hydrogenation of p-nitrophenol to p-aminophenol. *Chemosphere* **2022**, *291*, 132871.

[5] Zhou, J.; Zhang, Y.; Li, S.; Chen, J. Ni/NiO nanocomposites with rich oxygen vacancies as high-performance catalysts for nitrophenol hydrogenation. *Catalysts* **2019**, *9*(11), 944.

[6] Yan, Y.; Wu, J.; Wang, J.; Xu, M.; Zhou, W.; Li, Y.; Li, H. Rationally engineering magadiite heavy metal adsorbent for p-nitrophenol hydrogenation reduction. *Applied Clay Science* **2023**, *245*, 107143.

[7] Zhang, M.; Long, H.; Liu, Q.; Sun, L.; Qi, C. Synthesis of stable and highly efficient Au@ ZIF-8 for selective hydrogenation of nitrophenol. *Nanotechnology* **2020**, *31*(48), 485707.

[8] Liu, J.; Li, J.; Meng, R.; Jian, P.; Wang, L. Silver nanoparticles-decorated-Co_3O_4 porous sheets as efficient catalysts for the liquid-phase hydrogenation reduction of p-Nitrophenol. *Journal of colloid and interface science* **2019**, *551*, 261-269.

[9] Wei, F.; Luo, M.; Lan, J.; Xie, F.; Cai, L.; Chan, T. S.; Peng, Y. W.; Tan, Y. Pd Atomic Engineering of Nanoporous Ni/NiO for Efficient Nitrophenol Hydrogenation Reaction. *ACS Applied Materials & Interfaces* **2023**, *15*(22), 26746–26754

[10] Guan, H.; Chao, C.; Kong, W.; Hu, Z.; Zhao, Y.; Yuan, S.; Zhang, B. Magnetic porous PtNi/SiO_2 nanofibers for catalytic hydrogenation of p-nitrophenol. *Journal of Nanoparticle Research* **2017**, *19*, 1-11.
